# A Novel Role of the TRPM4 Ion Channel in Exocytosis

**DOI:** 10.3390/cells11111793

**Published:** 2022-05-30

**Authors:** Paulina Stokłosa, Sven Kappel, Christine Peinelt

**Affiliations:** Institute of Biochemistry and Molecular Medicine, University of Bern, 3012 Bern, Switzerland; paulina.stoklosa@ibmm.unibe.ch (P.S.); sven.kappel@ibmm.unibe.ch (S.K.)

**Keywords:** TRPM4, exocytosis, cancer cell

## Abstract

Under physiological conditions, the widely expressed calcium-activated TRPM4 channel conducts sodium into cells. This sodium influx depolarizes the plasma membrane and reduces the driving force for calcium entry. The aberrant expression or function of TRPM4 has been reported in various diseases, including different types of cancer. TRPM4 is mainly localized in the plasma membrane, but it is also found in intracellular vesicles, which can undergo exocytosis. In this study, we show that calcium-induced exocytosis in the colorectal cancer cell line HCT116 is dependent on TRPM4. In addition, the findings from some studies of prostate cancer cell lines suggest a more general role of TRPM4 in calcium-induced exocytosis in cancer cells. Furthermore, calcium-induced exocytosis depends on TRPM4 ion conductivity. Additionally, an increase in intracellular calcium results in the delivery of TRPM4 to the plasma membrane. This process also depends on TRPM4 ion conductivity. TRPM4-dependent exocytosis and the delivery of TRPM4 to the plasma membrane are mediated by SNARE proteins. Finally, we provide evidence that calcium-induced exocytosis depends on TRPM4 ion conductivity, not within the plasma membrane, but rather in TRPM4-containing vesicles.

## 1. Introduction

The transient receptor potential melastatin 4 (TRPM4) channel is a widely expressed calcium-activated nonselective (CAN) channel, which conducts monovalent cations such as sodium (Na^+^) [1]. It is directly activated via an increase in intracellular calcium (Ca^2+^), but it is not permeable to Ca^2+^ [2]. TRPM4-mediated Na^+^ influx depolarizes the plasma membrane and reduces the driving force for Ca^2+^ entry [3]. A TRPM4-mediated decrease in Ca^2+^ entry has been observed in various cellular systems, including immune, cancer, cardiac muscle, and dental pulp cells [1,3,4,5,6,7,8,9,10,11]; however, other groups have found that TRPM4 mediates an increase in Ca^2+^ entry [12,13,14]. The aberrant expression or malfunction of TRPM4 has been characterized in multiple diseases, such as cardiovascular [15,16,17], neurological [18], and skin diseases [19], as well as in different types of cancer [14,20,21,22,23,24,25,26,27,28,29,30,31,32,33]. Furthermore, TRPM4 plays a role in various immune cell functions [3,11,34,35].

TRPM4 is not only localized in the plasma membrane but also in intercellular vesicles [36]. The translocation of TRPM4 within intracellular vesicles has been associated with the process of insulin secretion in pancreatic β-cells [37], smooth muscle cell depolarization, the vasoconstriction of cerebral arteries [38], and the regulation of the antigen-evoked rise in Ca^2+^ in connective-tissue-type mast cells [39]. However, no studies have demonstrated that TRPM4 activity contributes directly to exocytosis. 

Evidence for the role of ion channels in regulated exocytosis has been reported in studies of different cells, such as the secretory cells of the pancreas, parietal cells, chromaffin cells, and gastric epithelial cells. In these cells, ion channels are involved in the secretion of different molecules via the regulation of exocytosis [37,40,41,42,43]. There are many ion channels that have been identified as being specific for the endolysosomal compartment, which include TRPML channels and P2X4, TMEM175, BK, CLCs, TRPM2, and TPC channels. These ion channels contribute to the regulation of lysosomal functions, such as lysosome movement, membrane trafficking, nutrient-sensing, membrane repair, organelle membrane contact, and lysosome biogenesis and adaptation [44,45,46,47]. Additionally, ion channels from the TRP family are involved in the regulation of exocytosis. TRPM7 is located in the membrane of acetylcholine (ACh)-secreting synaptic vesicles in sympathetic neurons, where it mediates neurotransmitter release [48]. Furthermore, TRPM7 has been shown to be located in ACh-secreting small synaptic-like vesicles in PC12 cells, where its ion conductivity across the vesicle membrane, rather than its enzymatic activity, has been shown to play a role in vesicle fusion [49]. Another TRP channel, TRPML1, has been shown to be a lysosomal Ca^2+^ channel that regulates focal exocytosis, which is required for phagosome formation during phagocytosis in macrophages [50]. Finally, the trafficking of some TRP channels to the plasma membrane, i.e. TRPM8 and TRPV1, has been shown to be dependent on SNARE (soluble *N*-ethylmaleimide-sensitive factor attachment protein receptors) proteins [51,52], which are transmembrane proteins that are essential for the fusion of lipid bilayers [53].

Previously, we have shown that TRPM4 is the main source of Ca^2+^-activated Na^+^ entry in the colorectal cancer (CRC) cell line HCT116 and that TRPM4 ion conductivity is involved in the regulation of cellular functions, such as cell viability and cell cycle in different CRC cells [29,32]. TRPM4 is controlled by p53, and the loss of p53 function in late-stage CRC cancer may enhance the role of TRPM4 in CRC progression [54]. Here, we report that TRPM4 is involved in the regulation of Ca^2+^-induced exocytosis in CRC cells. In this study, we aimed to elucidate whether Ca^2+^-induced exocytosis depends on TRPM4 activity and the specific mechanism of this process. 

## 2. Materials and Methods

### 2.1. Cell Culture

All cell lines were cultured at 37 °C, under 5% CO_2_, and at a relative humidity of 95%, according to the American Type Cell Culture Collection (ATCC, Rockville, MD, USA) guidelines. M4KO and M4KO cells with stable expressions of M4WT, M4D984A, and the GFP control construct were generated previously [32]. Cells were passaged every few days with the treatment of trypsin/EDTA. All cell culture work was conducted under sterile conditions. 

The human colorectal carcinoma cell line HCT116, originally derived from Dukes’ stage A CRC, was a gift from Karen Rother [55]. HCT116 and M4KO 1–5 cells were cultivated in McCoy’s 5A (Modified) medium with GlutaMAX (Gibco, #36600021), supplemented with 10% FBS (Gibco, #10270106). For the cultivation of M4KO with the stable expression of M4WT, M4D984A, and the GFP control construct, McCoy’s 5A (modified) medium with GlutaMAX, supplemented with 10% FBS and 0.25 μg/mL puromycin (Gibco, #A1113803), was used.

Lymph node carcinoma of the prostate (LNCaP) cells, derived from androgen-sensitive prostate cancer cells and DU145 and PC3 (both derived from androgen-insensitive prostate cancer) were purchased from the ATCC and cultured in RPMI medium 1640 (Life Technologies, Carlsbad, CA, USA), supplemented with 10% FCS and 1% penicillin/streptomycin (Life Technologies) [4].

### 2.2. Transient Transfection of HCT116 Cells with Tetanus Toxin Light Chain

For the expression of tetanus toxin light chain, 4 × 10^5^ HCT116 cells per well were seeded 24 h before transfection in 6-well plates (for the patch clamp experiments), or at a density of 3.5 × 10^5^ cells per 10 cm^2^ plates (for the biotinylation assay). The confluence of the cells was approximately 80% at the time of transfection. The plasmids used included: TetX-LC-pCAGG-IRES-GFP (tetanus toxin light chain, TeNT) [56] and pCAGGS-IRES-GFP (control). The transfection was performed with the FuGENE transfection kit (Promega, #E2311) according to the manufacturer’s instructions. The ratio of the FuGENE reagent to DNA was 4:1, and FuGENE and the DNA were diluted using Opti-MEM medium (Gibco, #31985062). For the transfection of cells in 6-well plates, 2 µg of DNA construct per well was used, and for the transfection of cells in 10 cm^2^ plates, 8 µg of DNA was used. After 15 min of incubation, 100 µL of the DNA/FuGENE/media mixture was added to each well of the 6-well plate, or 400 µL was added in the case of the 10 cm^2^ plates. For the patch clamp experiments, cells were re-seeded 24 h after transfection on 35 mm cell culture dishes, and recordings were performed 48 h after transfection (only GFP-positive cells were analyzed). The biotinylation assay was performed 48 h post-transfection.

### 2.3. Electrophysiology

Patch clamp experiments were performed in a tight-seal whole-cell configuration at 22–25 °C. The patch pipettes had resistances of between 2 and 3 MΩ. The current recordings were acquired with the patch clamp amplifier system EPC 10 (HEKA). After establishing the whole-cell configuration, voltage ramps of 50 ms duration, spanning from −100 to +100 mV or from −100 to 0 mV, from a holding potential of 0 mV, were delivered every 2 s (0.5 Hz). The capacitive currents were determined and corrected before each voltage ramp delivery. All voltages were corrected for a liquid junction potential of 10 mV. The currents were filtered at 1 kHz, and the sample rate was 3 kHz. For the analysis, the currents were extracted at −80 mV and 80 mV, normalized to the cell capacitance, and plotted versus time. The bath solutions contained (in mM): 140 NaCl, 0.5 CaCl_2_, 3 MgCl_2_, and 10 HEPES. The pH was adjusted with NaOH to 7.2. In the *N*-methyl-d-glucamine (NMDG) bath solution, 140 mM NaCl was replaced by 140 mM NMDG. The pH of the NMDG bath solution was adjusted to 7.2 with HCl. The osmolarity of both bath solutions was adjusted with glucose to around 300 mOsm. The internal solution contained (in mM): 140 Cs glutamate, 10 HEPES, 8 NaCl, 1 EGTA, MgCl_2_, and CaCl_2_. The MgCl_2_ and CaCl_2_ concentrations were calculated (https://somapp.ucdmc.ucdavis.edu/pharmacology/bers/maxchelator/webmaxc/webmaxcS.htm (accessed on 27 July 2019)) and added to reach a final concentration of 3 mM free Mg^2+^ and 10 µM free Ca^2+^. The data analysis and the graphical presentation of the patch clamp experiments were completed with Igor Pro 6.37 software (Wavemetrics).

### 2.4. Biotinylation Assay

For the biotinylation assay, 4 × 10^6^ cells were seeded onto 10 cm^2^ plates. To activate TRPM4, the cells were treated with ionomycin or DMSO as a control for 15 min at 37 °C. Then, the cells were put on ice and washed twice with ice-cold PBS, and treated with 5 mL of freshly prepared EZ-link Sulfo-NHS-SS-Biotin (Thermo Scientific, #21331) in cold 1 × PBS solution (0.5 mg/mL) for 15 min at 4 °C. To inactivate the biotin and to remove the cell debris, the cells were washed twice with 200 mM glycine in cold 1 × PBS, and then twice with cold 1 × PBS. Next, the total protein lysate was isolated with M-PER buffer (Thermo Scientific, #78501) supplemented with 1 × Halt Protease Inhibitor Cocktail (Thermo Scientific, #87786) and 1 × benzonase. The protein concentrations of the lysates were measured with the Pierce BCA Protein Assay Kit. Streptavidin Sepharose High Performance resin (50 µL) was washed twice with 500 µL of lysis buffer (2 min at 3000 rpm, 4 °C). Approximately 500 µg of each protein lysate was incubated with 50 µL of Streptavidin resin for 2 h at 4 °C on a wheel. Afterwards, the samples were washed five times with lysis buffer and eluted with 50 μL of 2 × NuPAGE sample buffer supplemented with 100 mM DTT at 37 °C for 30 min. Finally, the samples were centrifuged for 2 min at 3000 rpm and 4 °C, and the supernatant was kept at −20 °C for further analysis.

The plasma membrane protein was detected with Western blot. Additionally, the input fraction (whole-cell protein expression) was analyzed as a reference. Proteins were separated on 10% SDS-PAGE gel, and the electrophoresis was run for approximately 40 min at 200 V. The proteins were transferred to a nitrocellulose membrane for 1.5 h at a constant current of 350 mA at 4 °C. The membranes were blocked for 2 h with 3% BSA in TBS at room temperature. The proteins were probed with the following primary antibodies: rabbit anti-TRPM4 antibody [57], mouse anti-α Na^+^/K^+^-ATPase antibody (Abcam, #ab7671), and mouse anti-β-actin (Cell Signaling, #3700), overnight at 4 °C. Afterwards, the membranes were washed three times for 10 min with TBST buffer (Tween^®^ 20) and incubated with the fluorescent antibodies IRDye donkey anti-mouse (LI-COR, #925-68022) and IRDye 800CW goat anti-rabbit (LI-COR, #925-32211) for 1.5 h at room temperature. After incubation, the membranes were washed three times with TBST. The fluorescence of the antibodies was detected with the LI-COR Odyssey Imaging System and quantified with LI-COR Image Studio Lite software. For the membrane fraction, the α Na^+^/K^+^-ATPase expression level was used as a loading control, and β-actin was used as a negative control. 

## 3. Results

### 3.1. Exocytosis in HCT116 Cells Is Dependent on TRPM4

**Figure 1:**
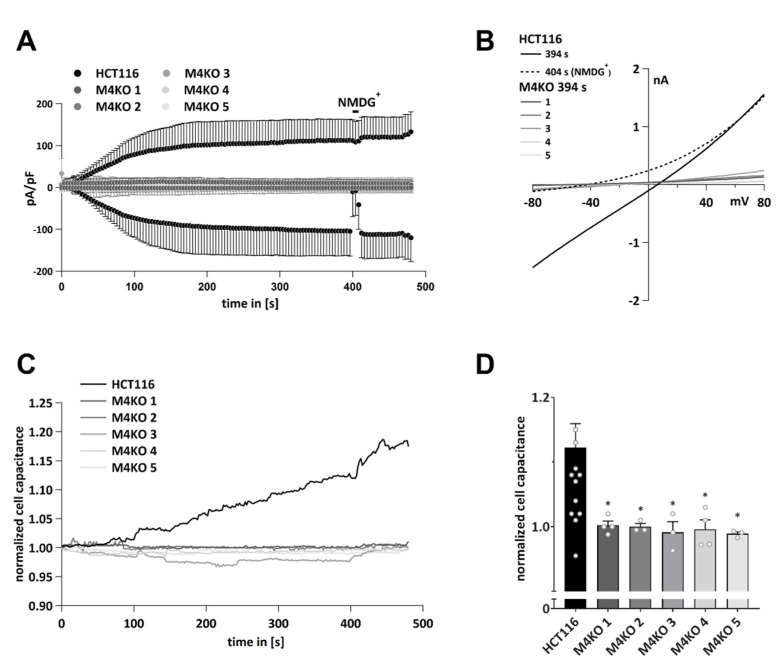
Patch clamp analysis and analysis of cell capacitance changes in HCT116 and M4KO clones. (**A**) I_CAN_ was evoked with 10 µM Ca^2+^ in the pipette (*n* = 14 for HCT116, *n* = 4 for M4KO 1, *n* = 3 for M4KO 2, *n* = 3 for M4KO 3, *n* = 3 for M4KO 4, and *n* = 3 for M4KO 5). (**B**) Corresponding averaged IVs at 394 s and 404 s (before and during application of NMDG^+^) from cells in (A). (**C**) Detected changes in cell capacitance in HCT116 and M4KO cell lines after activation with 10 µM Ca^2+^. The cell capacitance was normalized to the first point of each measurement and plotted versus time (*n* = 14 for HCT116, *n* = 4 for M4KO 1, *n* = 3 for M4KO 2, *n* = 3 for M4KO 3, *n* = 3 for M4KO 4, and *n* = 3 for M4KO 5). (**D**) Scatter plot and bar diagram of data in (**C**) at 394 s, shown as mean + SD. Mann–Whitney test for non-parametric data was used to determine statistical significance (* *p* < 0.05).

Upon the activation of HCT116 cells with 10 µM intracellular Ca^2+^, we detected high inward currents, which were completely abolished by the exchange of Na^+^ to NMDG^+^ in the bath solution, which suggests Na^+^ is the primary conducted ion (Figure 1A,B). These currents were completely abolished in the HCT116 TRPM4 knock-out cells, M4KO 1–5 (Figure 1A,B), which confirmed our previous results that identified TRPM4 as the main source of I_CAN_ (Ca^2+^-activated nonselective current) in the CRC cell line HCT116 [32]. During the whole-cell patch clamp measurements, we additionally detected the cell capacitance before each voltage ramp. The cell capacitance (measured in pF) is proportional to the cell surface area, and changes in cell capacitance reflect exocytosis (and/or endocytosis) [58,59]. An analysis of the HCT116 cells activated with 10 µM Ca^2+^ showed an increase in cell capacitance, which was absent in five M4KO cell lines (Figure 1C,D). Ca^2+^ is well known to induce regulated exocytosis [37,60]. In our experimental setup, the increase in intracellular Ca^2+^ triggered exocytosis, but only in cells expressing TRPM4. This leads to the conclusion that in HCT116, Ca^2+^-induced exocytosis is dependent on TRPM4.

### 3.2. TRPM4-Dependent Exocytosis in Prostate Cancer Cells

**Figure 2:**
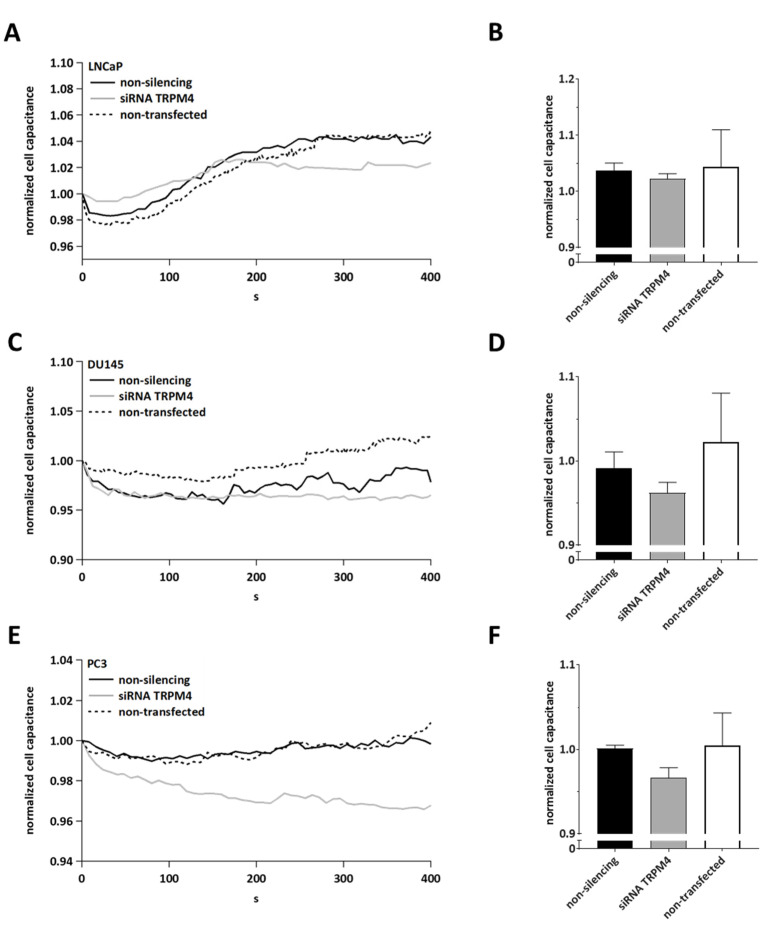
Patch clamp analysis and analysis of cell capacitance changes in PCa cells. (**A**) Detected changes in cell capacitance in LNCaP cells after activation with 10 µM Ca^2+^. The cell capacitance was normalized to the first point of each measurement and plotted versus time (*n* = 5 for non-silencing, *n* = 5 for siRNA TRPM4, and *n* = 9 for non-transfected). (**B**) Bar diagram of data in (A) at 394 s, shown as mean + SEM. (**C**) Detected changes in cell capacitance in DU145 cells after activation with 10 µM Ca^2+^. The cell capacitance was normalized to the first point of each measurement and plotted versus time (*n* = 5 for non-silencing, *n* = 7 for siRNA TRPM4, and *n* = 6 for non-transfected). (**D**) Bar diagram of data in (**C**) at 394 s, shown as mean + SEM. (**E**) Detected changes in cell capacitance in PC3 cells after activation with 10 µM Ca^2+^.The cell capacitance was normalized to the first point of each measurement and plotted versus time (*n* = 6 for non-silencing, *n* = 5 for siRNA TRPM4, and *n* = 9 for non-transfected). (**F**) Bar diagram of data in (**E**) at 394 s, shown as mean + SEM.

Next, we hypothesized whether the Ca^2+^-induced, TRPM4-dependent increase in cell capacitance is characteristic of CRC cells or whether there is a similar effect in prostate cancer (PCa) cell lines that also express high levels of TRPM4 [4,22]. To investigate this, we analyzed cell capacitance traces from previously recorded measurements published in [4] from the following PCa cell lines: LNCaP (Figure 2A,B), DU145 (Figure 2C,D), and PC3 (Figure 2E,F). These cell lines correspond to different PCa stages and were either non-transfected or transfected with siRNA targeting TRPM4 or control RNA. Under these conditions, the Ca^2+^-induced increase in cell capacitance was reduced upon the down-regulation of TRPM4 with siRNA in all three cell lines, suggesting a more general role for TRPM4 in Ca^2+^-induced cancer cell exocytosis. These changes in cell capacitance were not statistically significant; however, changes in cell capacitance may be an underestimation due to the incomplete down-regulation of TRPM4 by siRNA (~40% for LNCaP, ~57% for DU145, and ~71% for PC3 [4]), and the transfection may also affect changes in cell capacitance, which seems to be the case for DU145. 

### 3.3. TRPM4 Is Delivered to the Plasma Membrane upon the Increase in Intracellular Calcium

**Figure 3:**
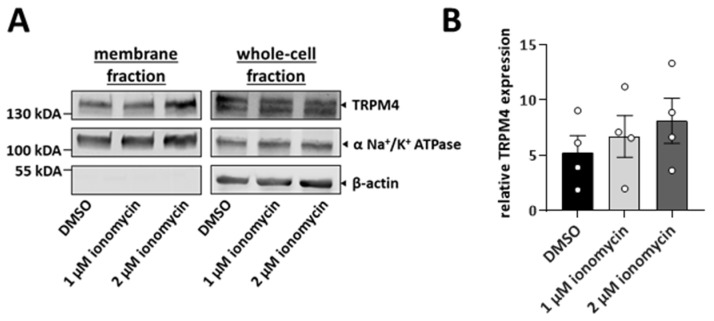
Increase in TRPM4 in the plasma membrane after stimulation with ionomycin. (**A**) HCT116 cells were stimulated with 1 or 2 µM ionomycin for 15 min, and TRPM4 membrane expression was analyzed using a biotinylation assay. DMSO was used as a control. TRPM4 expression in membrane and whole-cell fractions. For the membrane fraction, α-Na^+^/K^+^-ATPase was used as a loading control, and β-actin was used as a negative control. For the whole-cell fraction, β-actin was used as a loading control. (**B**) Scatter plot and bar diagram of the quantification of the TRPM4 plasma membrane expression levels, shown as mean + SD. Experiment was repeated four times.

TRPM4 is delivered to the plasma membrane via intracellular vesicles [36]. To elucidate whether an increase in intracellular Ca^2+^ can trigger the delivery of TRPM4 to the plasma membrane, we stimulated HCT116 cells with ionomycin and performed cell surface biotinylation assays. Ionomycin is a Ca^2+^ ionophore that transports Ca^2+^ to the cytosol and increases intracellular Ca^2+^. After 15 min of stimulation, either with 1 µM or 2 µM ionomycin, TRPM4 membrane expression increased in a dose-dependent manner (Figure 3). In conclusion, an increase in intracellular Ca^2+^ leads to the delivery of TRPM4-containing vesicles to the plasma membrane.

### 3.4. TRPM4 Ion Conductivity Plays a Role in Exocytosis

**Figure 4:**
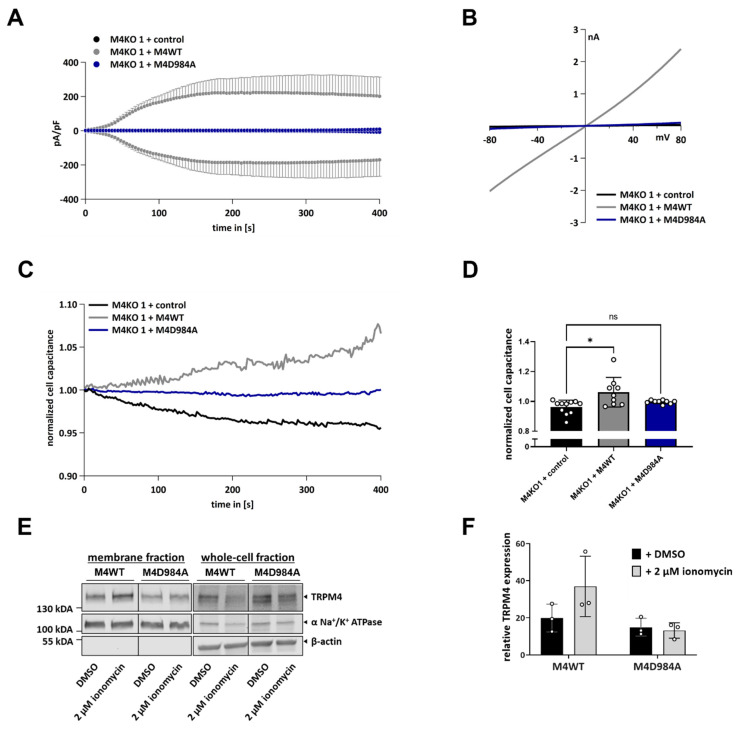
Analysis of M4KO 1 with expression of functional TRPM4 (M4WT) or dominant-negative TRPM4 mutant (M4D984A). (**A**) I_CAN_ was evoked with 10 µM Ca^2+^ in the pipette (*n* = 11 for M4KO 1 + control, *n* = 9 for M4KO 1 + M4WT, *n* = 9 for M4KO 1 + M4D984A). (**B**) Corresponding IVs at 394 s from cells in (A). (**C**) Normalized cell capacitance from cells in (A) after activation with 10 µM Ca^2+^ cell capacitance was plotted versus time (*n* = 12 for M4KO 1 + control, *n* = 9 for M4KO 1 + M4WT, and *n* = 9 for M4KO 1 + M4D984A). (**D**) Bar diagram of data in (**C**) at 394 s. Kruskal–Wallis test for non-parametric data was used to determine statistical significance (* *p* < 0.05, non-significant (ns)). (**E**) Plasma membrane and whole-cell expression of TRPM4 (M4WT or M4D984A). Cells were stimulated with 2 µM ionomycin or DMSO for 15 min, and TRPM4 membrane expression was analyzed with a biotinylation assay. DMSO was used as a control. α-Na^+^/K^+^-ATPase was used as a loading control for the membrane fraction, and β-actin was used as a loading control for whole-cell TRPM4 fraction. (**F**) Scatter plot and bar diagram of quantification of TRPM4 plasma membrane expression levels, shown as mean + SD. Experiment was repeated three times.

To investigate whether TRPM4 conductivity is necessary for Ca^2+^-induced exocytosis, we used HCT116 cells with TRPM4 knock-out, overexpressing either functional TRPM4 (M4KO 1 + M4WT) or a dominant-negative TRPM4 mutant (M4KO 1 + M4D984A), which has a mutation in the pore region of the channel and is unable to conduct any ions [2,32]. Whole-cell patch clamp measurements revealed that M4KO 1 cells, which express a functional TRPM4 channel, displayed high Na^+^ currents after their activation with Ca^2+^ in the patch pipette, while cells expressing the M4D984A mutant did not display the TRPM4-characteristic currents (Figure 4A,B). A cell capacitance analysis showed that only HCT116 cells expressing M4WT displayed an increase in cell capacitance after their activation with Ca^2+^ (Figure 4C,D). No changes were observed in cells expressing M4D984A, suggesting that TRPM4 conductivity plays a role in Ca^2+^-induced exocytosis. In HCT116, Ca^2+^-induced exocytosis is dependent on TRPM4 ion conductivity.

Next, we investigated whether TRPM4 ion conductivity is necessary for the delivery of TRPM4 to the plasma membrane, upon an increase in intracellular Ca^2+^. For this process, we stimulated M4KO 1 + M4WT and M4KO 1 + M4D984A with 2 µM ionomycin or a DMSO control for 15 min and performed cell-surface biotinylation assays (Figure 4E,F). Only in the M4KO 1 + M4WT, treatment with ionomycin resulted in an increase in plasma membrane TRPM4. In the M4KO 1 + M4D984A cells, TRPM4 plasma membrane expression did not change. These results suggest that TRPM4 ion conductivity is necessary for the Ca^2+^-induced delivery of TRPM4 to the plasma membrane.

### 3.5. TRPM4-Dependent Exocytosis Is Mediated by SNARE Proteins

**Figure 5:**
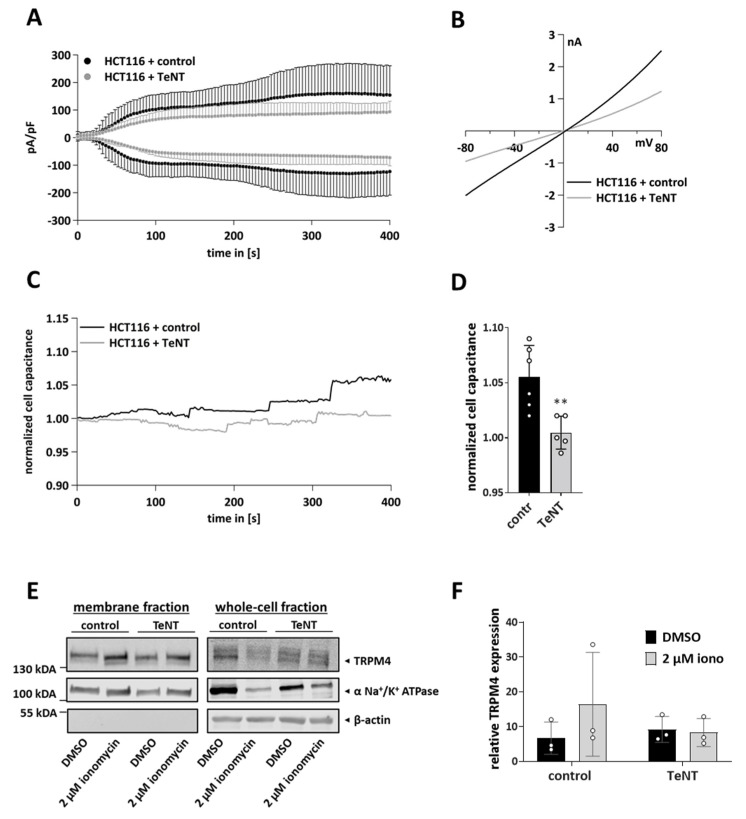
Overexpression of tetanus toxin in HCT116 cells. HCT116 cells were transfected with tetanus toxin light chain (TeNT) or the control vector. (**A**) Whole-cell patch clamp recordings were performed 48 h post-transfection. I_CAN_ was evoked with 10 µM Ca^2+^ in the pipette (*n* = 6 for HCT116 + control, *n* = 7 for HCT116 + TeNT). (**B**) Corresponding IVs at 394 s from cells in A. (**C**) Normalized cell capacitance plotted versus time for cells in A. (**D**) Bar diagram of data (mean + SEM) at 394 s from (**C**). Statistical analysis was performed with unpaired *t*-test (** *p* < 0.01). (**E**) Biotinylation assay was performed 48 h after transfection. Cells were stimulated with 2 µM ionomycin or DMSO for 15 min, and TRPM4 membrane expression was analyzed with a biotinylation assay (plasma membrane and whole-cell expression of TRPM4). DMSO was used as a control. α-Na^+^/K^+^-ATPase was used as a loading control for the membrane fraction and β-actin as a loading control for whole-cell TRPM4 fraction. (**F**) Quantification of TRPM4 plasma membrane expression levels, shown as mean + SEM from three independent experiments.

SNARE proteins are known to mediate exocytosis [53]. Here, we aimed to elucidate whether TRPM4-dependent exocytosis is dependent on these proteins. For this process, we overexpressed tetanus toxin light chain (TeNT) using a bicistronic vector [56]. Tetanus toxin light chain cleaves SNARE proteins, resulting in their deactivation [61]. We performed patch clamp measurements in cells expressing TeNT. Notably, cells with TeNT expression showed a tendency to exhibit decreased intensities of currents (Figure 5A,B); however, this decrease was not statistically significant. Cells transfected with the control vector showed an increase in cell capacitance, while cells expressing the tetanus toxin showed almost no change (Figure 5C,D). The expression levels of the tetanus toxin may have been insufficient to entirely block SNARE-mediated exocytosis. Next, we performed biotinylation assays in cells transfected with TeNT (Figure 5E,F). After stimulation with 2 µM ionomycin, cells transfected with the control vector showed an increase in the amount of membrane TRPM4. This increase was abrogated in cells transfected with TeNT. This suggests that TRPM4-dependent exocytosis and the delivery of TRPM4-containing vesicles to the plasma membrane are mediated by SNARE proteins.

### 3.6. TRPM4-Dependent Exocytosis Does Not Require Plasma Membrane TRPM4 Conductivity

**Figure 6:**
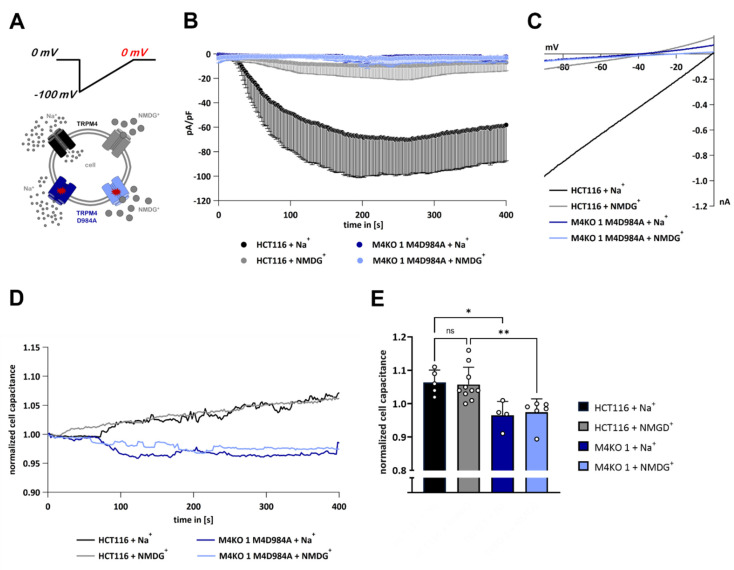
“Half-ramp” experiments in HCT116 and M4KO 1 cells. (**A**) Graphical representation of the experiment. Voltage ramps spanning from −100 to 0 mV were delivered from a holding potential of 0 mV; under these conditions, only an inward TRPM4 current occurred (upper panel). HCT116 cells endogenously expressing TRPM4 or M4KO 1 expressing M4D984 were used, and either Na^+^ or NMDG^+^ were present in the bath solution, to allow for or to prevent inward currents (lower panel). (**B**) I_CAN_ was evoked with 10 µM Ca^2+^ in the pipette (*n* = 5 for HCT116 + Na^+^, *n* = 10 for HCT116 + NMGD^+^, *n* = 4 for M4KO 1 M4D984A + Na^+^, and *n* = 6 for M4KO 1 M4D984A + NMDG^+^). (**C**) Corresponding IVs at 394 s from cells in (**B**). (**D**) Scatter plot and normalized cell capacitance plotted versus time from cells in (**B**). (**E**) Scatter plot and bar diagram of data (mean + SD) at 394 s from (**D**). Kruskal–Wallis test for non-parametric data was used to determine statistical significance (* *p* < 0.05, ** *p* < 0.01, and non-significant (ns)).

Next, we hypothesized whether TRPM4-dependent exocytosis is dependent on the activity of TRPM4 in the plasma membrane or within the vesicles. We used cells expressing either endogenous TRPM4 (HCT116) or solely the non-conducting TRPM4 mutant D984A (M4KO 1 M4D984A). Cells were activated with 10 µM Ca^2+^ in the patch pipette. We performed whole-cell patch clamp measurements and delivered voltage ramps spanning only negative potentials from −100 mV to 0 mV (“Half-ramp” experiments, Figure 6A, upper panel). At these negative membrane potentials, only the TRPM4 inward currents could occur, while the outward currents were avoided. Furthermore, we placed the cells in a bath solution containing either Na^+^ to allow for inward currents through activated TRPM4 channels or NMDG^+^ to prevent inward currents. An overview of our experimental conditions is given in Figure 6A (lower panel). 

When endogenous TRPM4 was expressed and Na^+^ was present in the bath solution, inward Na^+^ currents were observed, while in the NMDG^+^ bath solution, no inward currents were detected (Figure 6B, IVs in Figure 6C). In both conditions, we observed an increase in cell capacitance, shown in Figure 6D,E, demonstrating that the increase in cell capacitance was independent of TRPM4 conductivity in the plasma membrane. To investigate whether the increase in cell capacitance depended on TRPM4 conductivity in the vesicles, we performed the same experiments with M4KO 1 + M4D984A cells (Figure 6B–E). In these cells, when either Na^+^ or NMDG^+^ was present in the bath solution, no increase in cell capacitance was observed. This demonstrates that the presence of non-conducting TRPM4 in intracellular vesicles is insufficient to mediate exocytosis. In summary, TRPM4-dependent exocytosis is independent from plasma membrane TRPM4, but depends on TRPM4 conductivity in intracellular vesicles.

## 4. Discussion

We demonstrated that TRPM4 plays a role in Ca^2+^-induced exocytosis in the colorectal cancer cell line HCT116. An investigation of the changes in cell capacitance in HCT116 cells and HCT116 cells with the TRPM4 knock-out (M4KO) revealed that the expression of TRPM4 is necessary for exocytosis, with this being reflected as an increase in cell capacitance. Interestingly, the capacitance increase only occurred in cells expressing functional TRPM4 (M4WT), but not in the dominant negative TRPM4 mutant (M4D984A). This suggests that Ca^2+^-induced exocytosis in HCT116 is dependent on TRPM4 conductivity. Furthermore, we showed that the conductivity of plasma membrane TRPM4 is not necessary for an increase in cell capacitance. Hence, we propose that the conductivity of TRPM4 within intracellular vesicles is necessary for exocytosis in HCT116 cells. Furthermore, we showed that TRPM4 is delivered to the plasma membrane upon a rise in intracellular Ca^2+^. Previous studies have shown that TRPM4 is present in dynamic vesicles [36], suggesting that TRPM4 is delivered to the plasma membrane upon activation with Ca^2+^, in a process that is dependent on TRPM4 conductivity. Moreover, our data suggest a more general role for TRPM4 in Ca^2+^-induced exocytosis in cancer cells with high levels of TRPM4 expression, such as PCa cells.

The overexpression of the tetanus toxin light chain in HCT116 cells resulted in an inhibition of Ca^2+^-induced exocytosis. Furthermore, the overexpression of the tetanus toxin abrogated the Ca^2+^-induced delivery of TRPM4 to the plasma membrane. This leads to the conclusion that TRPM4-dependent exocytosis and the Ca^2+^-induced translocation of TRPM4 to the plasma membrane is mediated by SNARE proteins. These findings are in line with a previously reported mechanism of SNARE-dependent trafficking to the plasma membrane of other members of the TRP ion channel family, TRPV1 and TRPM8 [51,52]. Interestingly, cells expressing TeNT also showed reduced TRPM4-mediated currents. After activation with Ca^2+^, the delivery of TRPM4-containing vesicles to the plasma membrane may occur for the functional purpose of recruiting additional channels to further increase the Na^+^ influx. This increase in Na^+^ influx may then further reduce the driving force for Ca^2+^, and thus, fusion of TRPM4-containing vesicles to the plasma membrane may enhance the negative feedback mechanism for Ca^2+^ entry by membrane depolarization. Additionally, the control transfected HCT116 cells displayed a biphasic current after their activation with Ca^2+^. Similar observations were made in pancreatic β-cells, in which a dynamic translocation of TRPM4 to the plasma membrane via Ca^2+^- induced exocytosis resulted in biphasic currents [37]. This could support the hypothesis that an increase in intracellular Ca^2+^ concentration triggers the delivery of functional TRPM4 channels from the intracellular pool to the plasma membrane. However, such a conclusion is difficult to make, as we did not observe such a biphasic current in other whole-cell patch clamp experiments that investigated TRPM4 currents in HCT116 cells. 

Lysosomal exocytosis is a Ca^2+^-induced process that is mediated by SNARE proteins. This process involves the secretion of lysosome-associated membrane protein 1 (LAMP1)-containing vesicles [62]. It was shown that the exocytosis of LAMP1-containing vesicles might contribute to cancer features, including enhanced cell migration [63]. It was previously shown that TRPM4 mediates the migration of CRC and PCa cells [4,22,25,30,32]. Therefore, TRPM4-dependent exocytosis might be involved in the regulation of cancer cell migration and/or contribute to polarized exocytosis in metastasis [64,65]; however, further studies are needed to investigate that hypothesis. 

**Figure 7:**
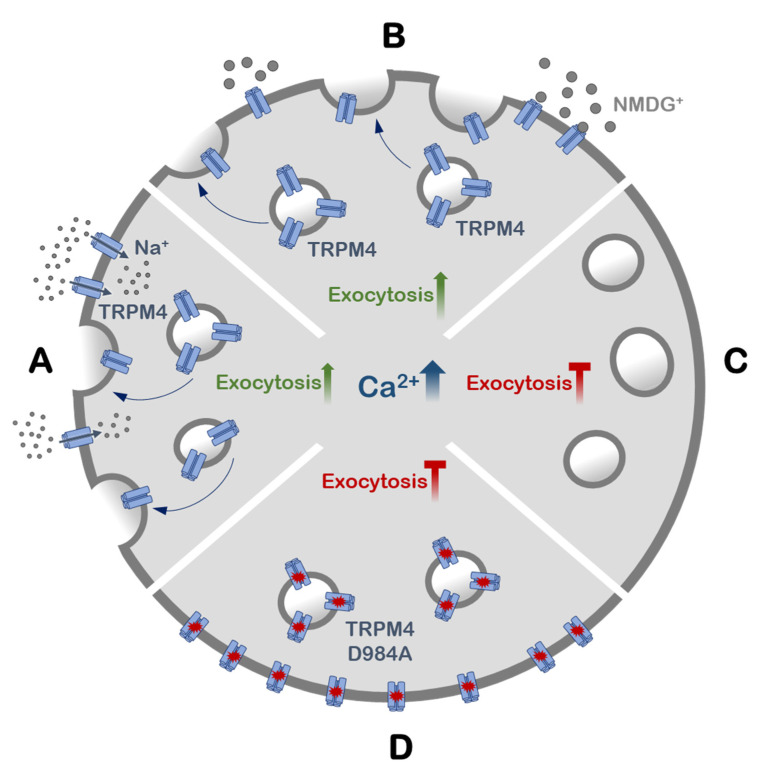
Graphical representation of the main findings. (**A**) An increase in intracellular Ca^2+^ triggers the exocytosis of TRPM4-containing vesicles. (**B**) Ca^2+-^ induced exocytosis is independent of plasma membrane TRPM4. (**C**) Exocytosis is inhibited when TRPM4 is absent. (**D**) Exocytosis is inhibited when non-functional TRPM4 is expressed.

Based on our findings, we propose a novel mechanistic model of TRPM4 contribution to Ca^2+^-induced exocytosis in HCT116 cells (a summary of the experiments is presented in Figure 7). After an increase in the intracellular Ca^2+^ concentration, TRPM4 channels are activated and they inwardly conduct Na^+^. Ca^2+^ also activates TRPM4 channels in the vesicular pool, and the conductivity of TRPM4 in this vesicular pool, rather than in the plasma membrane, results in the translocation of these vesicles and their fusion with the plasma membrane. 

## Data Availability

All data are available online (https://zenodo.org/record/6552390#.Yoc0OFTP2Uk (created on 16 May 2022)).

## References

[1] Launay P., Fleig A., Perraud A.L., Scharenberg A.M., Penner R., Kinet J.P. (2002). TRPM4 Is a Ca^2+^-Activated Nonselective Cation Channel Mediating Cell Membrane Depolarization. Cell.

[2] Nilius B., Prenen J., Janssens A., Owsianik G., Wang C., Zhu M.X., Voets T. (2005). The Selectivity Filter of the Cation Channel TRPM4. J. Biol. Chem..

[3] Launay P., Cheng H., Srivatsan S., Penner R., Fleig A., Kinet J.P. (2004). TRPM4 Regulates Calcium Oscillations after T Cell Activation. Science.

[4] Holzmann C., Kappel S., Kilch T., Jochum M.M., Urban S.K., Jung V., Stöckle M., Rother K., Greiner M., Peinelt C. (2015). Transient Receptor Potential Melastatin 4 Channel Contributes to Migration of Androgen-Insensitive Prostate Cancer Cells. Oncotarget.

[5] Kecskés M., Jacobs G., Kerselaers S., Syam N., Menigoz A., Vangheluwe P., Freichel M., Flockerzi V., Voets T., Vennekens R. (2015). The Ca^2+^-Activated Cation Channel TRPM4 Is a Negative Regulator of Angiotensin II-Induced Cardiac Hypertrophy. Basic Res. Cardiol..

[6] Kilch T., Kappel S., Peinelt C. (2016). Regulation of Ca^2+^ Signaling in Prostate Cancer Cells. Channels.

[7] Barbet G., Demion M., Moura I.C., Serafini N., Léger T., Vrtovsnik F., Monteiro R.C., Guinamard R., Kinet J.-P., Launay P. (2008). The Calcium-Activated Nonselective Cation Channel TRPM4 Is Essential for the Migration but Not the Maturation of Dendritic Cells. Nat. Immunol..

[8] Ngoc Tran T.D., Stovall K.E., Suantawee T., Hu Y., Yao S., Yang L.-J., Adisakwattana S., Cheng H. (2017). Transient Receptor Potential Melastatin 4 Channel Is Required for Rat Dental Pulp Stem Cell Proliferation and Survival. Cell Prolif..

[9] Diszházi G., Magyar Z.É., Lisztes E., Tóth-Molnár E., Nánási P.P., Vennekens R., Tóth B.I., Almássy J. (2021). TRPM4 Links Calcium Signaling to Membrane Potential in Pancreatic Acinar Cells. J. Biol. Chem..

[10] Weber K.S., Hildner K., Murphy K.M., Allen P.M. (2010). Trpm4 Differentially Regulates Th1 and Th2 Function by Altering Calcium Signaling and NFAT Localization. J. Immunol..

[11] Vennekens R., Olausson J., Meissner M., Bloch W., Mathar I., Philipp S.E., Schmitz F., Weissgerber P., Nilius B., Flockerzi V. (2007). Increased IgE-Dependent Mast Cell Activation and Anaphylactic Responses in Mice Lacking the Calcium-Activated Nonselective Cation Channel TRPM4. Nat. Immunol..

[12] Cantero-Recasens G., Butnaru C.M., Brouwers N., Mitrovic S., Valverde M.A., Malhotra V. (2019). Sodium Channel TRPM4 and Sodium/Calcium Exchangers (NCX) Cooperate in the Control of Ca^2+^-Induced Mucin Secretion from Goblet Cells. J. Biol. Chem..

[13] Fliegert R., Glassmeier G., Schmid F., Cornils K., Genisyuerek S., Harneit A., Schwarz J.R., Guse A.H. (2007). Modulation of Ca^2+^ Entry and Plasma Membrane Potential by Human TRPM4b. FEBS J..

[14] Sagredo A.I., Sagredo E.A., Cappelli C., Báez P., Andaur R.E., Blanco C., Tapia J.C., Echeverría C., Cerda O., Stutzin A. (2018). TRPM4 Regulates Akt/GSK3-β Activity and Enhances β-Catenin Signaling and Cell Proliferation in Prostate Cancer Cells. Mol. Oncol..

[15] Palladino A., Papa A.A., Petillo R., Scutifero M., Morra S., Passamano L., Nigro V., Politano L. (2022). The Role of TRPM4 Gene Mutations in Causing Familial Progressive Cardiac Conduction Disease: A Further Contribution. Genes.

[16] Ozhathil L.C., Rougier J.-S., Arullampalam P., Essers M.C., Ross-Kaschitza D., Abriel H. (2021). Deletion of Trpm4 Alters the Function of the Na(v)1.5 Channel in Murine Cardiac Myocytes. Int. J. Mol. Sci..

[17] Dienes C., Kovács Z.M., Hézső T., Almássy J., Magyar J., Bányász T., Nánási P.P., Horváth B., Szentandrássy N. (2021). Pharmacological Modulation and (Patho)Physiological Roles of TRPM4 Channel-Part 2: TRPM4 in Health and Disease. Pharmaceuticals.

[18] Schattling B., Steinbach K., Thies E., Kruse M., Menigoz A., Ufer F., Flockerzi V., Brück W., Pongs O., Vennekens R. (2012). TRPM4 Cation Channel Mediates Axonal and Neuronal Degeneration in Experimental Autoimmune Encephalomyelitis and Multiple Sclerosis. Nat. Med..

[19] Wang H., Xu Z., Lee B.H., Vu S., Hu L., Lee M., Bu D., Cao X., Hwang S., Yang Y. (2019). Gain-of-Function Mutations in TRPM4 Activation Gate Cause Progressive Symmetric Erythrokeratodermia. J. Investig. Dermatol..

[20] Zhu L., Miao B., Dymerska D., Kuswik M., Bueno-Martínez E., Sanoguera-Miralles L., Velasco E.A., Paramasivam N., Schlesner M., Kumar A. (2022). Germline Variants of CYBA and TRPM4 Predispose to Familial Colorectal Cancer. Cancers.

[21] Borgström A., Peinelt C., Stokłosa P. (2021). TRPM4 in Cancer—A New Potential Drug Target. Biomolecules.

[22] Borgström A., Hauert B., Kappel S., Zoni E., Kiener M., Stokłosa P., Baur R., Spahn M., Kruithof-de Julio M., Peinelt C. (2021). Small Molecular Inhibitors Block TRPM4 Currents in Prostate Cancer Cells, with Limited Impact on Cancer Hallmark Functions. J. Mol. Biol..

[23] Berg K.D., Soldini D., Jung M., Dietrich D., Stephan C., Jung K., Dietel M., Vainer B., Kristiansen G. (2016). TRPM4 Protein Expression in Prostate Cancer: A Novel Tissue Biomarker Associated with Risk of Biochemical Recurrence Following Radical Prostatectomy. Virchows Arch..

[24] Armisén R., Marcelain K., Simon F., Tapia J.C., Toro J., Quest A.F.G., Stutzin A. (2011). TRPM4 Enhances Cell Proliferation through Up-Regulation of the β-Catenin Signaling Pathway. J. Cell. Physiol..

[25] Hong X., Yu J.-J. (2019). MicroRNA-150 Suppresses Epithelial-Mesenchymal Transition, Invasion, and Metastasis in Prostate Cancer through the TRPM4-Mediated β-Catenin Signaling Pathway. Am. J. Physiol. Cell Physiol..

[26] Wong K.K., Hussain F.A. (2020). TRPM4 Is Overexpressed in Breast Cancer Associated with Estrogen Response and Epithelial-Mesenchymal Transition Gene Sets. PLoS ONE.

[27] Verigos J., Kordias D., Papadaki S., Magklara A. (2021). Transcriptional Profiling of Tumorspheres Reveals TRPM4 as a Novel Stemness Regulator in Breast Cancer. Biomedicines.

[28] Stokłosa P., Borgström A., Kappel S., Peinelt C. (2020). TRP Channels in Digestive Tract Cancers. Int. J. Mol. Sci..

[29] Stokłosa P., Borgström A., Hauert B., Baur R., Peinelt C. (2021). Investigation of Novel Small Molecular TRPM4 Inhibitors in Colorectal Cancer Cells. Cancers.

[30] Sagredo A.I., Sagredo E.A., Pola V., Echeverría C., Andaur R., Michea L., Stutzin A., Simon F., Marcelain K., Armisén R. (2019). TRPM4 Channel Is Involved in Regulating Epithelial to Mesenchymal Transition, Migration, and Invasion of Prostate Cancer Cell Lines. J. Cell. Physiol..

[31] Loo S.K., Ch’ng E.S., Md Salleh M.S., Banham A.H., Pedersen L.M., Møller M.B., Green T.M., Wong K.K. (2017). TRPM4 Expression Is Associated with Activated B Cell Subtype and Poor Survival in Diffuse Large B Cell Lymphoma. Histopathology.

[32] Kappel S., Stokłosa P., Hauert B., Ross-Kaschitza D., Borgström A., Baur R., Galván J.A., Zlobec I., Peinelt C. (2019). TRPM4 Is Highly Expressed in Human Colorectal Tumor Buds and Contributes to Proliferation, Cell Cycle, and Invasion of Colorectal Cancer Cells. Mol. Oncol..

[33] Çoban G., Yildiz P., Doğan B., Şahin N., Gücin Z. (2021). Expression of Transient Receptor Potential Melastatin 4 in Differential Diagnosis of Eosinophilic Renal Tumors. Mol. Clin. Oncol..

[34] Shimizu T., Owsianik G., Freichel M., Flockerzi V., Nilius B., Vennekens R. (2009). TRPM4 Regulates Migration of Mast Cells in Mice. Cell Calcium.

[35] Bianchi B., Smith P.A., Abriel H. (2018). The Ion Channel TRPM4 in Murine Experimental Autoimmune Encephalomyelitis and in a Model of Glutamate-Induced Neuronal Degeneration. Mol. Brain.

[36] Ghosh D., Segal A., Voets T. (2014). Distinct Modes of Perimembrane TRP Channel Turnover Revealed by TIR-FRAP. Sci. Rep..

[37] Cheng H., Beck A., Launay P., Gross S.A., Stokes A.J., Kinet J.-P., Fleig A., Penner R. (2007). TRPM4 Controls Insulin Secretion in Pancreatic Beta-Cells. Cell Calcium.

[38] Crnich R., Amberg G.C., Leo M.D., Gonzales A.L., Tamkun M.M., Jaggar J.H., Earley S. (2010). Vasoconstriction Resulting from Dynamic Membrane Trafficking of TRPM4 in Vascular Smooth Muscle Cells. Am. J. Physiol. Cell Physiol..

[39] Rixecker T., Mathar I., Medert R., Mannebach S., Pfeifer A., Lipp P., Tsvilovskyy V., Freichel M. (2016). TRPM4-Mediated Control of FcεRI-Evoked Ca^2+^ Elevation Comprises Enhanced Plasmalemmal Trafficking of TRPM4 Channels in Connective Tissue Type Mast Cells. Sci. Rep..

[40] Thévenod F. (2002). Ion Channels in Secretory Granules of the Pancreas and Their Role in Exocytosis and Release of Secretory Proteins. Am. J. Physiol. Physiol..

[41] Song P., Groos S., Riederer B., Feng Z., Krabbenhöft A., Smolka A., Seidler U. (2009). KCNQ1 Is the Luminal K+ Recycling Channel during Stimulation of Gastric Acid Secretion. J. Physiol..

[42] Song P., Groos S., Riederer B., Feng Z., Krabbenhöft A., Manns M.P., Smolka A., Hagen S.J., Neusch C., Seidler U. (2011). Kir4.1 Channel Expression Is Essential for Parietal Cell Control of Acid Secretion. J. Biol. Chem..

[43] Dernick G., Alvarez de Toledo G., Lindau M. (2003). Exocytosis of Single Chromaffin Granules in Cell-Free inside-out Membrane Patches. Nat. Cell Biol..

[44] Sterea A.M., Almasi S., El Hiani Y. (2018). The Hidden Potential of Lysosomal Ion Channels: A New Era of Oncogenes. Cell Calcium.

[45] Li P., Gu M., Xu H. (2019). Lysosomal Ion Channels as Decoders of Cellular Signals. Trends Biochem. Sci..

[46] Grimm C., Chen C.-C., Wahl-Schott C., Biel M. (2017). Two-Pore Channels: Catalyzers of Endolysosomal Transport and Function. Front. Pharmacol..

[47] Gerndt S., Chen C.-C., Chao Y.-K., Yuan Y., Burgstaller S., Scotto Rosato A., Krogsaeter E., Urban N., Jacob K., Nguyen O.N.P. (2020). Agonist-Mediated Switching of Ion Selectivity in TPC2 Differentially Promotes Lysosomal Function. eLife.

[48] Krapivinsky G., Mochida S., Krapivinsky L., Cibulsky S.M., Clapham D.E. (2006). The TRPM7 Ion Channel Functions in Cholinergic Synaptic Vesicles and Affects Transmitter Release. Neuron.

[49] Brauchi S., Krapivinsky G., Krapivinsky L., Clapham D.E. (2008). TRPM7 Facilitates Cholinergic Vesicle Fusion with the Plasma Membrane. Proc. Natl. Acad. Sci. USA.

[50] Samie M., Wang X., Zhang X., Goschka A., Li X., Cheng X., Gregg E., Azar M., Zhuo Y., Garrity A.G. (2013). A TRP Channel in the Lysosome Regulates Large Particle Phagocytosis via Focal Exocytosis. Dev. Cell.

[51] Ghosh D., Pinto S., Danglot L., Vandewauw I., Segal A., Van Ranst N., Benoit M., Janssens A., Vennekens R., Vanden Berghe P. (2016). VAMP7 Regulates Constitutive Membrane Incorporation of the Cold-Activated Channel TRPM8. Nat. Commun..

[52] Camprubí-Robles M., Planells-Cases R., Ferrer-Montiel A. (2009). Differential Contribution of SNARE-Dependent Exocytosis to Inflammatory Potentiation of TRPV1 in Nociceptors. FASEB J. Off. Publ. Fed. Am. Soc. Exp. Biol..

[53] Jahn R., Fasshauer D. (2012). Molecular Machines Governing Exocytosis of Synaptic Vesicles. Nature.

[54] Kappel S., Ross-Kaschitza D., Hauert B., Rother K., Peinelt C. (2022). P53 Alters Intracellular Ca^2+^ Signaling through Regulation of TRPM4. Cell Calcium.

[55] Rother K., Johne C., Spiesbach K., Haugwitz U., Tschöp K., Wasner M., Klein-Hitpass L., Möröy T., Mössner J., Engeland K. (2004). Identification of Tcf-4 as a Transcriptional Target of P53 Signalling. Oncogene.

[56] Zhang J., Castle D. (2011). Regulation of Fusion Pore Closure and Compound Exocytosis in Neuroendocrine PC12 Cells by SCAMP1. Traffic.

[57] Ozhathil L.C., Delalande C., Bianchi B., Nemeth G., Kappel S., Thomet U., Ross-Kaschitza D., Simonin C., Rubin M., Gertsch J. (2018). Identification of Potent and Selective Small Molecule Inhibitors of the Cation Channel TRPM4. Br. J. Pharmacol..

[58] Carrillo L., Cucu B., Bandmann V., Homann U., Hertel B., Hillmer S., Thiel G., Bertl A. (2015). High-Resolution Membrane Capacitance Measurements for Studying Endocytosis and Exocytosis in Yeast. Traffic.

[59] Houy S., Martins J.S., Mohrmann R., Sørensen J.B. (2021). Measurements of Exocytosis by Capacitance Recordings and Calcium Uncaging in Mouse Adrenal Chromaffin Cells. Methods Mol. Biol..

[60] Barclay J.W., Morgan A., Burgoyne R.D. (2005). Calcium-Dependent Regulation of Exocytosis. Cell Calcium.

[61] Sweeney S.T., Broadie K., Keane J., Niemann H., O’Kane C.J. (1995). Targeted Expression of Tetanus Toxin Light Chain in Drosophila Specifically Eliminates Synaptic Transmission and Causes Behavioral Defects. Neuron.

[62] Rao S.K., Huynh C., Proux-Gillardeaux V., Galli T., Andrews N.W. (2004). Identification of SNAREs Involved in Synaptotagmin VII-Regulated Lysosomal Exocytosis. J. Biol. Chem..

[63] Machado E., White-Gilbertson S., van de Vlekkert D., Janke L., Moshiach S., Campos Y., Finkelstein D., Gomero E., Mosca R., Qiu X. (2015). Regulated Lysosomal Exocytosis Mediates Cancer Progression. Sci. Adv..

[64] Kajiho H., Kajiho Y., Frittoli E., Confalonieri S., Bertalot G., Viale G., Di Fiore P.P., Oldani A., Garre M., Beznoussenko G.V. (2016). RAB2A Controls MT1-MMP Endocytic and E-cadherin Polarized Golgi Trafficking to Promote Invasive Breast Cancer Programs. EMBO Rep..

[65] Zeng J., Feng S., Wu B., Guo W. (2017). Polarized Exocytosis. Cold Spring Harb. Perspect. Biol..

